# A Case Report of Ruptured Popliteal Aneurysm in the Setting of Blunt Trauma

**DOI:** 10.5811/cpcem.2022.10.57671

**Published:** 2023-01-19

**Authors:** Jaymes A.J. Lonzanida, Bryan E. Love, Brian L. Anderson

**Affiliations:** * Desert Regional Medical Center, Department of Emergency Medicine, Palm Springs, California; † Desert Regional Medical Center, Desert Trauma Surgeons, Palm Springs, California

**Keywords:** case report, popliteal artery, aneurysm rupture

## Abstract

**Introduction:**

Popliteal artery aneurysms are in most cases asymptomatic but cause significant complications if ruptured. An acute popliteal aneurysm rupture is relatively rare, and few cases have been documented secondary to blunt trauma. Common presenting signs and symptoms include distal limb ischemia and absent dorsalis pedis pulses. Timely management and recognition of this rare presentation are crucial as this condition can result in limb loss or death if not treated in a timely manner.

**Case Report:**

An 80-year-old man with history of hypertension presented to the emergency department complaining of inability to feel sensation below his left knee after falling from ground level. Physical examination was pertinent for bounding radial and femoral pulses bilaterally, although absent dorsalis pedis and posterior tibial pulses to the left lower extremity. Computed tomography angiography identified occlusion of the left superficial femoral arterial lumen associated with a ruptured popliteal aneurysm, approximately eight centimeters in size. He immediately received unfractionated heparin and was admitted to the hospital for left medial thigh exploration and decompressive dermatofasciotomy.

**Conclusion:**

After confirmation of popliteal aneurysmal rupture with advanced imaging, heparinization and vascular surgery consultation are critical steps that should be taken to prevent limb loss.

## INTRODUCTION

Popliteal artery aneurysms are in most cases asymptomatic but cause significant complications if ruptured. The incidence of popliteal aneurysm rupture is rare, estimated at 1% in males aged 65–80 years.^1^ To date, 58 cases of popliteal artery aneurysm rupture have been described in the literature, and only seven have been documented secondary to trauma.^2^,^3^ We present an acute ruptured popliteal aneurysm secondary to a fall from ground level.

## CASE REPORT

An 80-year-old man with history of hypertension presented to the emergency department (ED) complaining of inability to feel sensation below his left knee after falling from ground level. Prior to arrival, the prehospital paramedics reported loss of palpable pulses in the left lower extremity. Vital signs upon arrival included a blood pressure of 110/80 millimeters of mercury, heart rate of 110 beats per minute, 16 respirations per minute, an oxygen saturation of 98% on room air, and a temperature of 98.6° Fahrenheit. Physical examination was pertinent for the left lower extremity rotated externally in plantarflexion with tenderness and ecchymosis to the left medial thigh. Vascular examination revealed radial and femoral pulses bilaterally, although absent dorsalis pedis and posterior tibial pulse to the left lower extremity. Bedside arterial duplex revealed left posterior tibial monophasic waveforms and presence of diastolic flow.

**Image 1:**
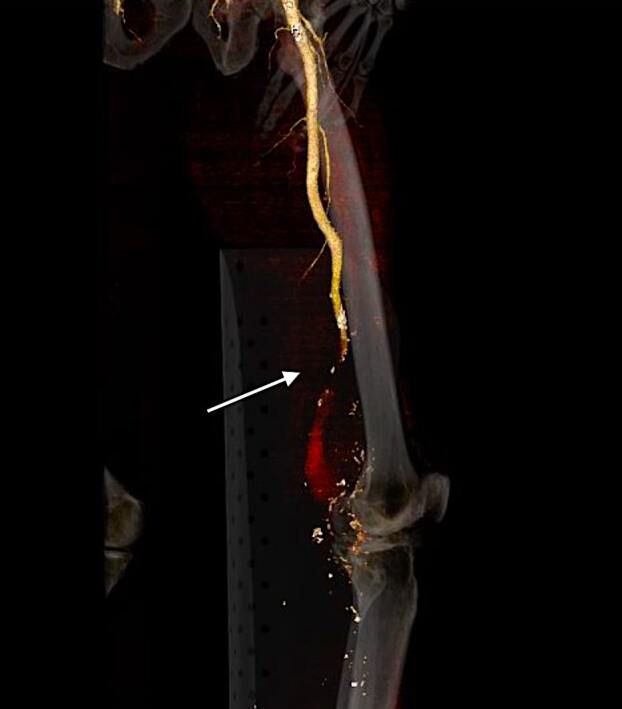
Computed tomography angiography volume-rendering technique reconstruction of the left lower extremity in sagittal view demonstrating superficial artery occlusion with popliteal artery aneurysm rupture (arrow) and lack of arterial contrast enhancement distally.

**Image 2:**
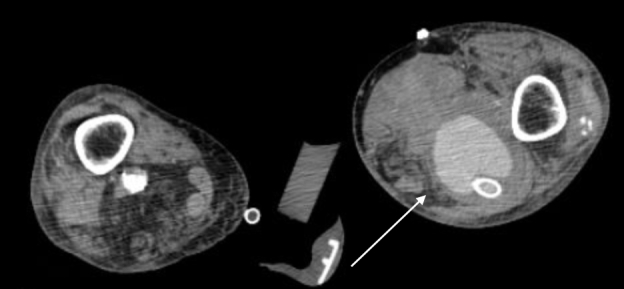
Computed tomography angiography of the left lower extremity in axial view demonstrating popliteal artery aneurysm extension into a large left lower leg hematoma (arrow) status post popliteal artery stenting and dermatofasciotomy.

Radiographs of his femur and left tibia were negative for acute fracture or bony abnormality. Computed tomography angiography of the left lower extremity with contrast (Images 1 and 2) identified compression of the superficial femoral arterial lumen with complete occlusion distally, associated with a ruptured popliteal aneurysm, approximately eight centimeters in size. In consultation with our vascular surgeon and trauma service, the patient was started on a heparin infusion and transferred to the operating room for left medial thigh exploration and decompressive dermatofasciotomy.

## DISCUSSION

Our patient was diagnosed with an acute ruptured popliteal aneurysm, likely secondary to blunt trauma after falling from ground level. The incidence of popliteal aneurysm rupture is rare, estimated at 1% in males aged 65–80 years.^1^ To date, 58 cases of popliteal artery aneurysm rupture have been described in the literature, and only seven have been documented secondary to trauma.^2^,^3^ Recognition of a ruptured popliteal artery aneurysm is the most difficult aspect in management, as ischemic signs may be absent, leading to alternative diagnoses, such as venous thrombosis, ruptured synovial cysts, and soft tissue sarcomas.^5^ The most common signs and symptoms of a ruptured popliteal aneurysm, regardless of mechanism, are similar to those of compartment syndrome and include a swollen extremity, acute lower limb ischemia, paresthesias, and nerve deficits.^5^ Timely management and recognition of aneurysmal rupture are vital because of the threat to limb loss and loss of life secondary to hemorrhagic shock.^3^

*CPC-EM Capsule*

What do we already know about this clinical entity?

*Popliteal artery aneurysms are commonly complicated by thrombosis and distal embolization.*

What makes this presentation of disease reportable?

*Popliteal artery aneurysm rupture is rare and few cases have been documented secondary to blunt trauma.*

What is the major learning point?

*Management of popliteal aneurysm rupture is facilitated with a detailed physical examination, appropriate radiographic studies, and expeditious consultation with specialists.*

How might this improve emergency medicine practice?

*Improved recognition and management of popliteal aneurysm rupture may decrease morbidity and mortality.*

When performing a primary assessment to an extremity involved in trauma, emergency physicians must assess sensation and motor function as paucity of either may indicate acute limb ischemia. When evaluating for popliteal aneurysms, the knee should be examined in a semi-flexed position, as 60% of patients with popliteal artery aneurysms have a palpable pulsatile mass at the level of the knee joint. Furthermore, the clinician must recognize the time elapsed from the insult as limb ischemia may be secondary to compartment syndrome. Nevertheless, if there is a high index of suspicion for acute limb ischemia, initial management with systemic unfractionated heparin should be prioritized to prevent aneurysmal thrombus propagation. Although no guidelines are available to guide management, most ruptured popliteal artery aneurysms are repaired surgically with rates of endovascular repair on the rise.^2^ Nevertheless, the condition may lead to amputation and is associated with a high risk of death within the first year after surgical repair.^3^

## CONCLUSION

Our patient likely had localized trauma to his knee, causing popliteal aneurysm rupture, hemorrhage, and compartment syndrome. He was immediately treated with heparin and admitted to the hospital for left medial thigh exploration, dermatofasciotomy, and left superficial femoral artery and popliteal artery stenting. His hospital course was complicated by proximal stent migration into the aneurysmal sac with thrombus causing a large hematoma approximately 46 centimeters in size. Incidentally, he was found to have a three-centimeter popliteal aneurysm in his contralateral right leg, which had not ruptured suggesting that his left popliteal aneurysm was a chronic issue prior to his insult. Unfortunately, despite pharmacologic and vascular stenting interventions, the patient died secondary to cardiac arrest. Although ruptured popliteal artery aneurysm is a rare event, management in the ED is facilitated with a detailed physical examination, appropriate radiographic studies, and expeditious consultation with specialists. Recognizing popliteal aneurysm rupture, utilization of advanced imaging, and surgical intervention are mainstays to decrease morbidity and mortality.
